# Supplementary material for: An integrated personal and population-based Egyptian genome reference
Source: Nat Commun. 2020 Sep 18;11:4719. doi: 10.1038/s41467-020-17964-1 (PMC7501257; doi:10.1038/s41467-020-17964-1)
Supplement: Supplementary file 3 — Descriptions of Additional Supplementary Files [file 41467_2020_17964_MOESM3_ESM.pdf]

Descriptions of Additional Supplementary Files

Supplementary Data 1:

Description: Generated next-generation sequencing data utilized in this study. Genome coverage was computed using an estimated genome size of 3Gb.

Supplementary Data 2:

Description: Complete assembly characteristics and quality metrics for the final Egyptian meta assembly (EGYPT), the corresponding WTDBG2- and FALCON-based assemblies (EGYPT_wtdbg2 and EGYPT_falcon) and for comparison for a published assembly of a Korean (AK1) and Yoruba (YORUBA) individual. This is the extended QUAST-LG report, which is by default based on contigs of size >= 3000 bp, unless otherwise noted (e.g., "# contigs (>= 0 bp)" and "Total length (>= 0 bp)" include all contigs). Reference-based QUAST-LG measures use GRCh38. Metrics in Sections "Misassembly classification" and "Base level QV" have not been computed with QUAST-LG. Base level QV of AK1 has been computed based on error rate reported by AK1 authors.

Supplementary Data 3:

Description: For meta assembly construction, aligned regions of the falcon-based assembly have been added if they overlapped with gaps greater 800kb in the wtdbg2-based assembly. This table lists the gaps and the corresponding overlapping aligned segments. Some aligned falcon-based segments cover more than one gap and have been added to the wtdbg2 assembly only once, which amounts to overall 387 falcon-based contigs being added for the construction of a meta assembly.

Supplementary Data 4:

Description: Repeat annotation results. For different classes and types of repeats, the number of identified repeats, their combined length and the percent of sequence they cover is listed for the reference genome GRCh38, Yoruban assembly YORUBA, Korean assembly AK1 and wtdbg2- and falcon based Egyptian assemblies.

Supplementary Data 5:

Description: Summary of variant phasing and SV calling using 10X Genomics data and 10X Genomics software longranger. Please note that "snps_phased" also considers homozygous reference allele positions.

Supplementary Data 6:

Description: Runs of homozygosity in the EGYPT assembly individual.

Supplementary Data 7:

Description: Summary of individuals and sequencing data used for the population genome. Mean depth denotes the mean sequencing depth over approx. 19 million small variant positions. Libraries and lanes denotes the number of sequencing libraries and combined number of different sequencing lanes used. Source denotes the source of the raw sequencing data. Coverage is a broad classification into low and high as refered to by Pagani et al. Origin denotes the Egyptian geographic region from which the corresponding individuals originate, if available.

Supplementary Data 8:

Description: Mean and standard deviation of the number of SVs per individual after collapsing. Also the minimum and maximum number for the respective SV type is given.

Supplementary Data 9:

Description: Unique insertions (NUIs) which have a coverage of more than 5 in at least 10 of 110 Egyptian individuals when mapping such reads to the assembly, which couldn't be mapped to the reference genome or GATK bundle sequences. Given is for every NUI sequence start, end and length; alignment block start and end in GRCh38 and in EGYPT assembly; number of SNVs/indels within the NUI (see Suppl. Table 10 for a list of these variants); Sequence ID if significantly similar to NUI reported by PUBMED IDs 30072691, 28250455, or 28104618, resp. with corresponding alignment information.

Supplementary Data 10:

Description: Variants within the unique insertions of Suppl. Table 9, identified in 110 Egyptian individuals.

Supplementary Data 11:

Description: Individuals used for population genetics analyses annotated with source data set, continent, world subregion, country, population, and genotyping method. The individual ID is the ID of the source data set preceded with the dataset name.

Supplementary Data 12:

Description: Datasets used in population genetics analyses.

Supplementary Data 13:

Description: Population genetic characterization of the Egyptian population. Principal component analysis with n=5,429 individuals from populations world-wide. This HTML is an interactive version of Figure 2c). Coloring is according to continental regions and shading denotes subregions. Mouseover will display the subregion of an individual. Subregions to be displayed can be selected in the upper right panel.

Supplementary Data 14:

Description: High-confidence set of population-specific variants with MAF at least 5% in the Egyptian cohort and less than 1% in populations from 1000 Genomes, GnomAD and in TOPMED.

Supplementary Data 15:

Description: VEP annotation of four Egyptian-specific common variants with a CADD deleteriousness score of more than 20; ordered by chromosome and position.

Supplementary Data 16:

Description: Variants that are common in the Egyptian cohort and have no dbSNP rsID assigned together with annotations and predicted effects; ordered by chromosome and position.

Supplementary Data 17:

Description: Genes with significant haplotypic expression in the EGYPT individual, which have been computed by phASER.

Supplementary Data 18:

Description: GWAS catalog associations of 4008 analyzed tag SNPs, sorted primarily by GWAS catalog column 'MAPPED_TRAIT' and secondarily by chromosome and position. EUR_N_CHR/EGP_N_CHR Number of chromosmes in European/Egyptian cohort with alleles; EUR_ALT_AF/EGP_ALT_AF: European/Egyptian allele frequenc of alt allele; EUR_N_PROXY/EGP_N_PROXY: Number of proxy variants ; PROXY_SHARED: Number of proxy variants in European and Egyptian cohort; PROXY_EUR_ONLY/PROXY_EGP_ONLY: Number of proxy variants only in European/Egyptian cohort. Proxy variants are defined as variants in linkage disequilibrium of R2>=0.8.
