# Supplementary material for: An integrated personal and population-based Egyptian genome reference
Source: Nat Commun. 2020 Sep 18;11:4719. doi: 10.1038/s41467-020-17964-1 (PMC7501257; doi:10.1038/s41467-020-17964-1)
Supplement: Supplementary file 22 — Reporting Summary [file 41467_2020_17964_MOESM22_ESM.pdf]

nature research

Corresponding author(s): Saleh Ibrahim & Hauke Busch

Last updated by author(s): 23/06/2020

Reporting Summary

Nature Research wishes to improve the reproducibility of the work that we publish. This form provides structure for consistency and transparency in reporting. For further information on Nature Research policies, see our Editorial Policies and the Editorial Policy Checklist.

Statistics

For all statistical analyses, confirm that the following items are present in the figure legend, table legend, main text, or Methods section.

n/a	Confirmed	
☐	☒	The exact sample size (n) for each experimental group/condition, given as a discrete number and unit of measurement
☒	☐	A statement on whether measurements were taken from distinct samples or whether the same sample was measured repeatedly
☒	☐	The statistical test(s) used AND whether they are one- or two-sided<br>*Only common tests should be described solely by name; describe more complex techniques in the Methods section.*
☒	☐	A description of all covariates tested
☒	☐	A description of any assumptions or corrections, such as tests of normality and adjustment for multiple comparisons
☒	☐	A full description of the statistical parameters including central tendency (e.g. means) or other basic estimates (e.g. regression coefficient) AND variation (e.g. standard deviation) or associated estimates of uncertainty (e.g. confidence intervals)
☒	☐	For null hypothesis testing, the test statistic (e.g. F, t, r) with confidence intervals, effect sizes, degrees of freedom and P value noted<br>*Give P values as exact values whenever suitable.*
☒	☐	For Bayesian analysis, information on the choice of priors and Markov chain Monte Carlo settings
☒	☐	For hierarchical and complex designs, identification of the appropriate level for tests and full reporting of outcomes
☒	☐	Estimates of effect sizes (e.g. Cohen's d, Pearson's r), indicating how they were calculated

Our web collection on statistics for biologists contains articles on many of the points above.

Software and code

Policy information about availability of computer code

Data collection	Data sources are provided in section Data
Data analysis	Github repositories lied_egypt_genome, Egyptref and 2020_population_genetics with workflows and scripts used in this study are available under the buschlab Github account at https://github.com/orgs/buschlab.

For manuscripts utilizing custom algorithms or software that are central to the research but not yet described in published literature, software must be made available to editors and reviewers. We strongly encourage code deposition in a community repository (e.g. GitHub). See the Nature Research guidelines for submitting code & software for further information.

Data

Policy information about availability of data

All manuscripts must include a data availability statement. This statement should provide the following information, where applicable:

- Accession codes, unique identifiers, or web links for publicly available datasets
- A list of figures that have associated raw data
- A description of any restrictions on data availability

All summary data of the Egyptian genome reference are available at www.egyptian-genome.org, where also variant allele frequencies can be queried online. Raw sequencing data and variant data are available at EGA under study ID EGAS00001004303 (Data set IDs: EGAD00001006034, EGAD00001006035, EGAD00001006036, EGAD00001006037, EGAD00001006038, EGAD00001006039, EGAD00001006040). De novo assemblies EGYPT, EGYPT_wtdbg2 and EGYPT_falcon have been deposited at DDBJ/ENA/GenBank
under the accessions JABMBW000000000, JABMBX000000000, JABMBY000000000. The versions described in this paper are versions JABMBW010000000, JABMBX010000000 and JABMBY010000000, respectively.
Assemblies of a Korean and a Yoruba genome have been obtained from GenBank under assembly accession IDs GCA_001750385.2 [https://www.ncbi.nlm.nih.gov/

assembly/GCA_001750385.2] and GCA_001524155.4 [https://www.ncbi.nlm.nih.gov/assembly/GCA_001524155.4], respectively.
Raw sequencing data of Pagani et al.22 used in this study is available at EGA under data set IDs EGAD00001001372 and EGAD00001001380.
World-wide genotype data used for population genetics analyses has been compiled from five sources, and accession codes and/or links are provided in Supplementary Data 12.

Field-specific reporting

Please select the one below that is the best fit for your research. If you are not sure, read the appropriate sections before making your selection.

[x] Life sciences [ ] Behavioural & social sciences [ ] Ecological, evolutionary & environmental sciences

For a reference copy of the document with all sections, see nature.com/documents/nr-reporting-summary-flat.pdf

Life sciences study design

All studies must disclose on these points even when the disclosure is negative.

Sample size	For the de novo assembly and population genetics 110 individuals were included, 10 self-recruited and 100 from a public repository.<br>For the mitochondrial genome, 327 were included: 10 same, self-recruited individuals from above, 217 recruited to the study and 100 from the same public repository (same individuals).
Data exclusions	No data or individuals were excluded.
Replication	No replication was performed as none was needed. The study aim was to assemble a new reference genome for Egyptian population. For this only a small number of representative individuals was necessary.
Randomization	The individuals included in the study were randomly chosen from those fulfilling the inclusion criteria (see below)
Blinding	N/A.

Reporting for specific materials, systems and methods

We require information from authors about some types of materials, experimental systems and methods used in many studies. Here, indicate whether each material, system or method listed is relevant to your study. If you are not sure if a list item applies to your research, read the appropriate section before selecting a response.

Materials & experimental systems

n/a	Involved in the study	
[x]	[ ]	Antibodies
[x]	[ ]	Eukaryotic cell lines
[x]	[ ]	Palaeontology and archaeology
[x]	[ ]	Animals and other organisms
[ ]	[x]	Human research participants
[x]	[ ]	Clinical data
[x]	[ ]	Dual use research of concern

Methods

n/a	Involved in the study	
[x]	[ ]	ChIP-seq
[x]	[ ]	Flow cytometry
[x]	[ ]	MRI-based neuroimaging

Human research participants

Policy information about studies involving human research participants

Population characteristics	Egyptians, adults, randomly chosen from those fullfiling the recruitment criteria.
Recruitment	Subjects were recruited from healthy relatives escorting patients admitted to Mansoura University hospital. Those consented to join the study and who met the main selection criteria, i.e. they identified as Egyptians up to the third generation, were included. Medical history was taken to ascertain no history of chronic diseases, followed by a full clinical examination by a medial doctor alongside routine laboratory investigations (Liver and kidney function tests and complete blood count (CBC)).
Ethics oversight	This study was approved by the Mansoura Faculty of Medicine Institutional Review Board (MFM-IRB) Approval Number RP/15.06.62. All subjects gave written informed consent in accordance with the Declaration of Helsinki. This study and its results are in accordance with the Jena Declaration (https://www.uni-jena.de/unijenamedia/Universit%C3%A4t/Abteilung +Hochschulkommunikation/Presse/Jenaer+Erkl%C3%A4rung/Jenaer_Erklaerung_EN.pdf).

Note that full information on the approval of the study protocol must also be provided in the manuscript.
